# MCRS1 Expression Regulates Tumor Activity and Affects Survival Probability of Patients with Gastric Cancer

**DOI:** 10.3390/diagnostics12061502

**Published:** 2022-06-20

**Authors:** Liang-Han Wang, Chih-Chun Chang, Chiao-Yin Cheng, Yao-Jen Liang, Dee Pei, Jen-Tang Sun, Yen-Lin Chen

**Affiliations:** 1 Department of Emergency Medicine, Far Eastern Memorial Hospital, New Taipei 220, Taiwan; wlh0326@gmail.com (L.-H.W.); chiaoyin810406@gmail.com (C.-Y.C.); 2 Department of Clinical Pathology, Far Eastern Memorial Hospital, New Taipei 220, Taiwan; chihchun.chang1211@gmail.com; 3 Graduate Institute of Applied Science and Engineering, Fu-Jen Catholic University, New Taipei 242, Taiwan; 071558@mail.fju.edu.tw; 4 Division of Endocrinology and Metabolism, Department of Internal Medicine Fu Jen Catholic University Hospital, School of Medicine, College of Medicine, Fu-Jen Catholic University, New Taipei 242, Taiwan; peidee@gmail.com; 5 Department of Pathology, Tri-Service General Hospital, National Defense Medical Center, Taipei 114, Taiwan

**Keywords:** MCRS1, gastric cancer, microspherule protein 1, MSP58, proliferation

## Abstract

Gastric cancer is the fifth most common cancer worldwide and the third most common cause of cancer-related deaths. Surgery remains the first-choice treatment. Chemotherapy is considered in the middle and advanced stages, but has limited success. Microspherule protein 1 (MCRS1, also known as MSP58) is a protein originally identified in the nucleus and cytoplasm that is involved in the cell cycle. High expression of MCRS1 increases tumor growth, invasiveness, and metastasis. The mechanistic relationships between MCSR1 and proliferation, apoptosis, angiogenesis, and epithelial–mesenchymal transition (EMT) remain to be elucidated. We clarified these relationships using immunostaining of tumor tissues and normal tissues from patients with gastric cancer. High MCRS1 expression in gastric cancer positively correlated with Ki-67, Caspase3, CD31, Fibronectin, pAKT, and pAMPK. The hazard ratio of high MCRS1 expression was 2.44 times that of low MCRS1 expression, negatively impacting patient survival.

## 1. Introduction

Recent epidemiological studies have demonstrated a rise in the incidence of cancer in adults under the age of 50, raising global concern [1]. Gastric cancer is the fifth most common cancer in the world, and is the third most common cause of death due to cancer [2,3,4,5]. According to the guidelines of the American Cancer Society, the TNM system is mainly used for staging gastric cancer, where T stands for primary and represents the extent of the tumor, i.e., whether the tumor has grown into the gastric parietal layer or has reached nearby structures or organs; N represents whether it has invaded the lymph nodes; and M represents whether it has metastasized to other organs. The T, N, and M categories are determined and combined to correspond to the cancer stage from stage 0 to the fourth (last) stage. The lower the stage, the less the spread of cancer. Initially, clinical staging is performed through physical examination, ultrasound, computed tomography, and gastroscopy. Pathological staging can be performed following surgical resection. After chemotherapy or radiation therapy for tumor shrinkage has been performed, the cancer stage is reassessed before performing surgery to improve its success rate. This is called neoadjuvant therapy [6]. The five-year survival rate of gastric cancer, if diagnosed early, is 90–95%, but the proportion of patients who are diagnosed at this early disease stage is very small [7,8]. Existing treatment options for gastric cancer include surgery and chemotherapy (reserved for patients with advanced forms of the disease). To date, these treatment options have displayed suboptimal efficacy, warranting discovery of more effective treatment options [9]. Microspherule protein 1 (MCRS1, also known as MSP58) was originally found in the nucleus, and can regulate the transcription of rRNA genes [10,11]. MCRS1 is also found in the cytoplasm, participates in the cell cycle, and regulates the stability of the mitotic spindle [12,13]. In addition to participating in mitotic spindle assembly and mTOR signaling, *MCRS1* is an oncogene [14,15]. MCSR1 has been found to be overexpressed in neuroblastoma, lung cancer, gastric cancer, liver cancer, colorectal cancer, and kidney cancer, and is associated with increased tumor growth, invasion and metastasis [15,16,17,18,19,20]. In 2019, using two gastric cancer cell lines (BGC-823 and SGC-7901), Wang et al. found that suppressing MCSR1 expression can inhibit tumor activity [15]. Thus, increased MCRS1 expression is speculated to increase growth, invasiveness, and metastatic potential of tumors. However, the relationships between MCSR1 and proliferation, apoptosis, angiogenesis, epithelial–mesenchymal transition (EMT), and other related common proteins need to be further explored. In addition, in previous studies, the related studies of MCRS1 in gastric cancer were limited to cell lines. No experiments were performed through human tumor tissue. In this article, we will use immunostaining analysis of excised tissue from gastric cancer patients to better clarify the role of MCRS1 in tumors. This study seeks to investigate the aforementioned phenomena.

## 2. Materials and Methods

### 2.1. Patient Selection

We retrospectively selected patients diagnosed with gastric cancer at Cardinal Tien Hospital between 2000 and 2013. All selected patients had undergone conventional surgical resection and regional lymph node dissection. We excluded patients who had received chemotherapy or radiotherapy before resection. In total, 181 patients were included in this study. All clinical data, including histopathological reports, were reviewed, and the age, sex, lymph node metastasis, and TNM stage of each patient was recorded. All patient data were anonymized to protect patient identities. This research protocol was approved by the Institutional Review Board of Cardinal Tien Hospital.

### 2.2. Tissue Array

All tumor samples were immersed in formalin and embedded in paraffin blocks. Then, 5-μm-thick sections were cut consecutively from formalin-fixed, paraffin-embedded tissue for hematoxylin and eosin (H&E) staining to confirm the locations of tumors and non-tumor tissues. The representative parts of both tumor and non-tumor parts were selected and marked on the slides. After selecting appropriate positions, 2 mm diameter cores were removed from the paraffin blocks and embedded in a new block to construct the tissue array. Subsequently, the tissues were cut into 5 μm sections and immunostaining was performed.

### 2.3. Immunohistochemical Staining (IHC) and H Score

The tissue blocks were cut into sections for immunohistochemical stain. The immunohistochemical stain was performed using a Ventana BenchMark XT automated stainer (Ventana, Tucson, AZ, USA). Briefly, 5-μm-thick sections were cut consecutively from formalin-fixed, paraffin-embedded tissue. Sections were mounted on silanized slides and allowed to dry at room temperature. The Ventana BenchMark XT automated stainer performed deparaffinization and rehydratation of the slides. After a washing procedure with the supplied buffer, tissue sections were repaired for 40 min with ethylenediamine tetraacetic acid. The slides were again incubated with the primary antibody for 60 min at 37 °C. After three rinses in buffer, the slides were incubated with the secondary antibodies (Universal DAB Detection Kit, Ventana, Tucson, AZ, USA). Tissue staining was visualized with a DAB substrate chromogen solution. Slides were counterstained with hematoxylin, dehydrated, and mounted. Tissue sections were stained with the primary antibodies MCRS1, Ki-67, Caspase3, CD31, E-cadherin, N-cadherin, Fibronectin, phosphorylated-AKT (pAKT), pERK, pSTAT3 and pAMPK using the Ventana BenchMark XT automatic stainer (Ventana, Tucson, AZ, USA) for IHC (Table 1). Slides were reviewed by an experienced pathologist (Y-L CHEN) and immunostaining intensity was recorded as 0 for no staining, 1 for weak staining, 2 for medium staining, and 3 for strong staining. In addition, the staining percentages in each sample were recorded. H-score ranging from 0 to 300 was calculated by multiplying the staining intensity by the staining percentage of each sample (ex. H score 300 means staining intensity of 3+ and stained 100% on the sample; H score = 3 × 100).

### 2.4. Statistical Analysis

All statistical analyses were performed using SPSS software (version 20.0; SPSS Inc., Chicago, IL, USA). Chi-square analysis of categorical variables was used for comparison of high and low MCRS1 expression. Student’s *t*-test was used to compare differences in the H-scores of different variables. Correlation analysis was used to examine the relationships between protein markers of tumor proliferation, metastasis, invasion, and apoptosis, and MCRS1 expression. The hazard ratio probability was calculated based on high or low MCRS1 expression during overall survival. We used the Kaplan–Meier method to analyze and draw survival curves. All statistical tests were two-way, and results with *p*-value < 0.05 were considered statistically significant.

## 3. Results

This study used samples from 181 patients with gastric cancer, including 122 (67.4%) women and 59 (32.6%) men. The mean age of the patients was 71.1 years. Patients were divided by age into two groups (≥65 years (27.6%) and <65-years (72.4%)). Each group was subdivided into two groups based on whether they displayed low or high MCRS1 expression. A higher percentage (17%) of patients over 65 years of age displayed high MCRS1 expression and for those under 65 it was 10%. The proportion of well-differentiated tumors was 28.7%, and 71.3% were moderately differentiated. The high expression of MCRS1 accounted for 7.7% and 14.0% of the well-differentiated and moderately differentiated groups, respectively. The ratio of early stage (stage I and II) to late stage (stages III and IV) cancer was divided into 40.9% and 59.1%, respectively. About 14% of patients in stages III and IV also displayed elevated MCRS1 expression compared to patients in the earlier stages I and II (9.5%) (Table 2).

We immunostained tumor tissues to observe the interactions between MCRS1 and other common proteins involved in proliferation, apoptosis, angiogenesis, epithelial–mesenchymal transition (EMT), and tyrosine kinases. Correlation of MCRS1 with Ki-67, Caspase3, CD31, and pAkt was assessed using Pearson’s correlation test. MCRS1 demonstrated a strong positive correlation with Ki-67, as well as a moderate or weak correlation with Caspase3, CD31, and pAkt (Table 3).

**Figure 1:**
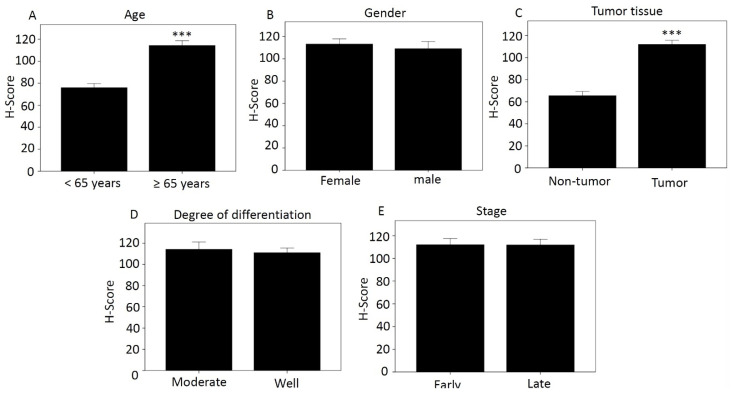
Differences in H-score of MCRS1 according to different categories. (**A**) Age; (**B**) Gender; (**C**) Tissue type; (**D**) Degree of differentiation; (**E**) Disease stage. *** *p*-value < 0.001.

Using the categories in Table 2, we compared H-score differences according to age (Figure 1A), sex (Figure 1B), tissue type (Figure 1C), degree of differentiation (Figure 1D), and disease stage (Figure 1E). Those aged over 65 years and with tumor tissue had higher H-score, 114.27 and 111.91, respectively, which was significant compared with less than 65 years and with non-tumor tissue. The H-scores for women and men were 113.25 and 109.16, respectively. The moderately differentiated score was 114.16, and the well-differentiated score was 111.01. The H-score was 112.02 for early-stage cancer and 111.84 for late-stage cancer.

**Figure 2:**
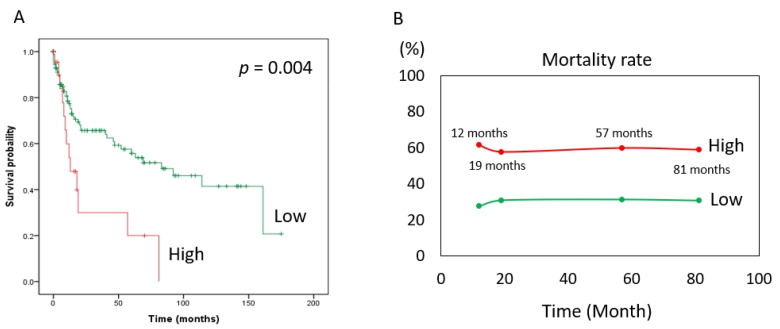
Kaplan–Meier plot of MCRS1. (**A**) The green line indicates low MCRS1 expression (N = 159). The red line indicates high expression of MCRS1 (N = 22). (*p*-value = 0.004) (**B**) Mortality at 12 months, 19 months, 57 months, and 81 months.

The risk of death (expressed as the hazard ratio) in the MCRS1 high expression group was 2.44 times that in the low expression group. In addition, according to our statistics, the MCRS1 high-expression group had a 2.44-fold higher risk of death than the low-expression group at the same time point, with a 95% CI of 1.31–4.57 and a *p* value of 0.005. The hazard ratio was 0.70 for patients older than 65 years compared with those younger than 65 years, with a 95% CI of 0.41–1.18. The hazard ratio for males to females was 0.74, 95% CI 0.42–1.32. The hazard ratio for well-differentiated versus moderate was 1.74, 95% CI 0.94–3.34, and age, sex, and degree of differentiation did not differ significantly in the risk of death at the same time. The hazard ratio for death at the same time was 2.50 in the later stage (III and IV) compared with the earlier stage (I and II) of the disease, with a 95% CI of 1.44–4.35, and the *p*-value was 0.001. Based on the above, we used Cox multivariate regression analysis and found that high expression of MCRS1 and advanced disease had a higher risk of death at the same time of onset. (Table 4). The Kaplan–Meier survival curve (Figure 2A) is consistent with the results shown in Table 4. The number of people with low expression of MCRS1 was 159, and the number of people with high expression of MCRS1 was 22. We listed the obvious turning points at 12 months, 19 months, 57 months, and 81 months, and presented them in Figure 2B, highlighting the mortality gap between high and low MCRS1 expression. High MCRS1 expression is associated with a higher risk of death and shorter survival time.

**Figure 3:**
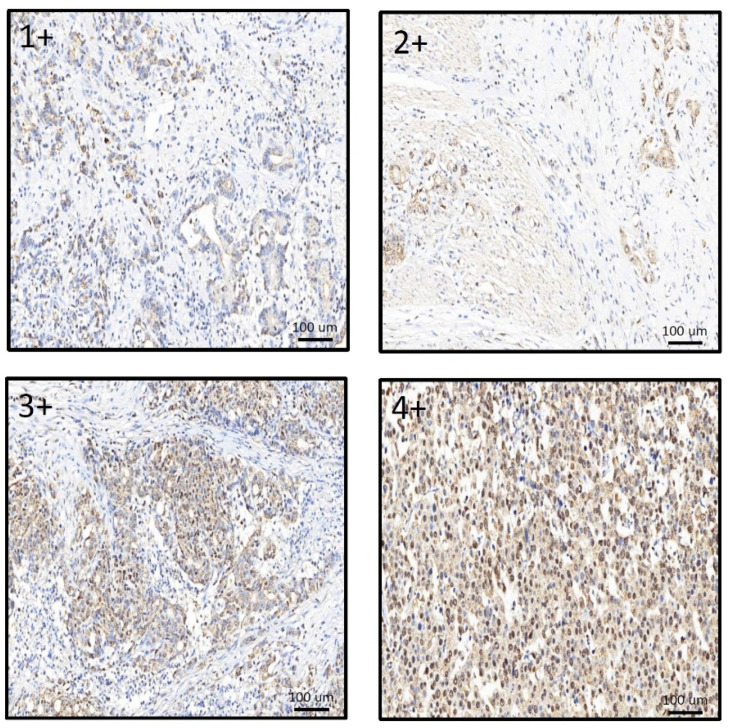
Comparison of different expression levels of MCRS1 in gastric tumor tissues.

**Figure 4:**
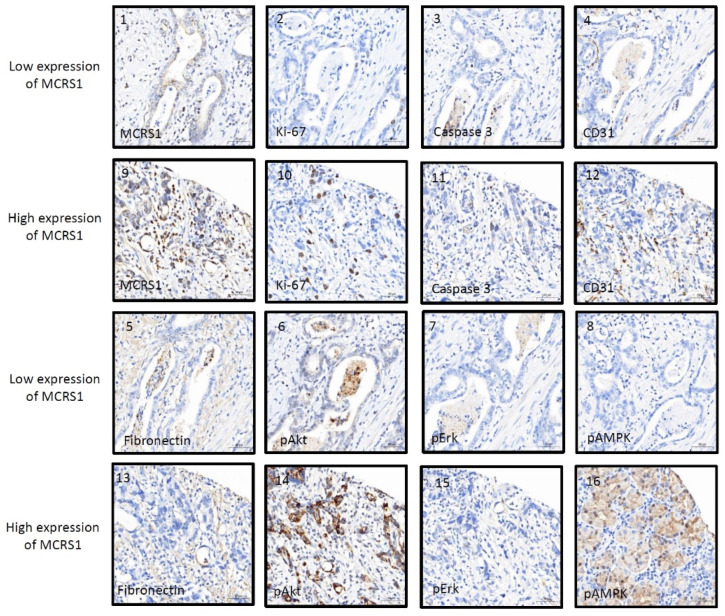
Immunostaining images of high or low expression levels of MCRS1 and related tumorigenic proteins. (1–8) Low MCRS1 expression levels. (9–16) High MCRS1 expression levels.

IHC staining was used to compare the relationship between MCRS1 expression and mortality risk. We scored different intensities of MCRS1 expression on a scale ranging from 1+ to 4+ (Figure 3) and calculated H-Scores. By selecting IHC staining at the same position of the tumor, we observed the level of MCRS1 expression, which affects the expression of Ki-67, Caspase3, fibronectin, pAKT, and pAMPK. Higher MCRS1 expression was associated with higher expression of the aforementioned proteins (Figure 4).

## 4. Discussion

In patients with gastric cancer, elevated MCSR1 is associated with increased mortality risk compared to lower MCRS1 expression. MCRS1 expression correlated with markers including Ki-67, Caspase3, CD31, fibronectin, pAKT, and pAMPK. Older patients with gastric cancer display higher levels of MCSR1 expression; however the underlying mechanism of this is unknown. Overexpression of MCRS1 in gastric cancer has been proposed to increase tumor proliferation, metastasis and invasion [21]. In addition to stomach cancer, similar results have been found in lung cancer, colorectal cancer, liver cancer, and kidney cancer [22,23,24,25]. Although there have been previous reports that MCRS1 is involved in gastric cancer proliferation, metastasis, and invasion, these reports are limited to cell experiments. This is the first study to use human tumor specimens to assess the expression of MCRS1 and other markers, and our findings confirm that MCRS1 is associated with Ki-67, Caspase3, CD31, fibronectin, pAKT, and pAMPK in gastric cancer [21].

In 55 patients with glioma, MCRS1 expression positively correlated with Ki-67, indicating a role for MCRS1 in tumor proliferation [26]. The Ki-67 protein is present in vertebrates and maps to human chromosome 10q26.2 with a total exon number of 16 [27]. The Ki-67 gene consists of two distinct protein isoforms, with alternative splicing of the mRNA precursor, with or without exon 7, with molecular weights of 320 and 359 kDa, respectively. The N-terminus of Ki-67 protein contains several domains, including a forkhead-associated domain, a protein phosphatase 1-binding domain, a large central region containing tandem repeats, and a C-terminal leucine/arginine-rich chromatin binding domain [28]. The human Ki-67 gene is encoded as MKI67, which contains 5 different isoforms, which share the same central tandem repeat sequence and C-terminal region [29,30]. In the G0 phase of the cell cycle, the expression level of Ki-67 is negligible. It increases from the late G1 phase to the S phase; and in the G1 phase, the expression level is increased by the regulation of E2F (cell cycle transcriptional regulator) and MCM2-MCM6 (replication-initiation complex protein minichromosome maintenance 2–6) [31,32]. The expression level is reduced by the regulation of the ubiquitin-proteasome complex APC/C-Cdh1. Ki-67 has been found to have prognostic value in many cancers, including lung, bladder, breast, and cervical cancers. However, high expression of Ki-67 in gastric cancer has not been associated with poor prognosis [27]. Ki-67 interacts with the nucleolar protein NIFK in the forkhead-associated domain to promote cell proliferation and cancer metastasis. NIFK destabilizes the transcription factor RUNX1 to enhance the metastatic ability of lung cancer cells, thereby stimulating the Wnt/β-catenin signaling pathway. The above-mentioned mechanism of action was confirmed in the lung cancer cell lines A549 and PC13 [33,34,35,36]. In addition, Ki-67 can be used in combination with other markers such as MCM-2, survivin, B7-H1, geminin, carbonic anhydrase, IX, gelsolin, MIB-1, and phosphorylated S6 protein, and the interaction between white (pS6) and vimentin affects the prognosis of renal cancer. Although we did not further explore the mechanism of Ki-67 in gastric cancer in this study, our experiments confirmed that the high expression of MCRS1 affects the prognosis of patients, and the influence of Ki-67 is also involved.

In lung cancer, liver cancer, and glioma cells, decreased MCRS1 expression can inhibit cell growth and increase the occurrence of apoptosis of cancer cells [26,37,38]. A few previous studies have examined the relationship between MCRS1 and Caspase 3. Our results show that MCRS1 positively correlates with Caspase 3, but further studies are needed to establish the underlying mechanism. Caspase 3 plays an important role in intracellular or extracellular apoptosis signaling pathways [39]. Caspase 3 and other caspase members are important proteins that mediate cell destruction during apoptosis. Caspases use positive or negative feedback mechanisms to regulate hormones, phosphorylation, or other proteins [40,41]. Although caspase 3 has focused on the apoptotic pathway in previous studies, an increasing number of studies have pointed out that caspase 3 is also involved in tumor growth. In 2011, the authors used caspase 3 in different states to verify whether caspase 3 promotes tumor growth. Breast cancer cell lines were generated using MCF7-expressing exogenous caspase 3, and 4T1 was generated by shRNA-mediated knockdown of caspase 3. The difference between the two was compared using irradiation. Compared to caspase 3-deficient cells, isogenic caspase 3 normal cells were significantly more effective at promoting irradiated cancer cell growth in vitro and tumor growth in vivo. Moreover, it was confirmed that caspase 3 promotes tumor growth through prostaglandin E2 downstream of the caspase 3-activated signaling pathway involving cytosolic calcium-independent phospholipase A 2 and arachidonic acid effects [42]. In addition to the destruction of caspase 3 itself, the interaction of caspase 3 and p21 in the DNA damage response also affects tumor growth [43]. In the DNA damage response, P21 protein is regulated by P53 protein, prompting cellular repair or initiation of apoptosis. Healthy P21 inhibits apoptosis; however, when P21 is cleaved into 15-KDa fragments, it promotes apoptosis. Caspase 3 mediates the production of a 15-KDa fragment from P21 that promotes apoptosis [44]. It was previously reported that the expression of caspase-3 is associated with poor and good prognosis in gastric cancer [45]. Our study indicates that the high expression of MCRS1 is positively correlated with the expression of Caspase 3. Although the mechanism has not been confirmed, it could be an interesting topic for future research.

Platelet/endothelial cell adhesion molecule 1 (PECAM-1), also known as CD31, has a molecular weight of 130 kDa [46]. CD31 is a marker of tumor metastasis in cancer diagnosis. CD31 induces hepatocellular carcinoma epithelial–mesenchymal transition through the ITGB1-FAK-Akt signaling pathway to regulate metastasis [47] and can also be used as a prognostic indicator for gastric cancer in the elderly population [48]. CD31 is involved in cell migration, survival, and formation of endothelial cell-connected tissues, and also has the function of maintaining the endothelial cell permeability barrier [49]. In experiments, the use of antibodies against CD31 causes endothelial cells grown on Matrigel to lose their ability to form tubular structures [50]. In CD31 knockout mice, tumors were also observed to show no signs of angiogenesis [51]. The regulation of angiogenesis by CD31 is carried out through the PECAM-1/SHP-2 complex and by altering the activity of the small G protein RhoA [52]. Endothelial cells are stimulated or inflamed, which can induce apoptosis, and are protected through homologous binding of CD31 and signaling through the CD31 cytoplasmic domain [53,54]. CD31 not only has important functions in cell migration and cell survival, but is also important for the maintenance of the endothelial cell permeability barrier. However, its detailed mechanism of action has not been described. CD31 can be used as a marker of lymphatic invasion [55]. In recent studies, an increasing number of people have regarded it as a prognostic marker. The prognosis of patients with colorectal cancer with high CD31 expression is poor [56]. Transgenic overexpression of CD31 accelerates subcutaneous JEKO1 and MINO tumor growth in immunodeficient mice [57]. However, high CD31 expression is not always an indicator of poor prognosis. Interestingly, in renal cell carcinoma, high expression of CD31 is significantly associated with better survival [58]. Our experiments revealed the increase in the expression of CD31 with the increased expression of MCRS1; hence, MCRS1 is positively correlated with CD31. The increased expression of MCRS1 causes decreased probability of survival in patients, although we could not confirm the detailed relationship.

EMT is a reversible cellular program that instantly transforms upper cells into a quasi-mesenchymal state [59]. EMT is regulated by EMT-inducible transcription factors (EMT-TFs), which exert pleiotropic and diverse combinatorial effects to induce gene expression that promotes the mesenchymal state and suppresses the maintenance of supraphysis state gene expression [60]. In the context of tumors, EMT programs regulated by EMT-TFs can be critical, resulting in the malignant progression of cancer cells, with promoted tumor-initiating properties, motility, dissemination ability, and increased resistance to cancer cell death [61,62]. Fibronectin is a mesenchymal cell biomarker [63]. It is composed of two monomers with a size of approximately 440 kDa [64]. Attieh et al. demonstrated that cellular fibronectin plays a role in carcinogenesis and invasion of tumor cells. In 2006, Zeng et al. reported that α5β1 signaling induced by fibronectin can rapidly activate focal adhesion kinase, leading to the generation of downstream invasive signaling [65]. Meng et al. demonstrated that inhibition of focal adhesion kinase activity abrogates the ability of fibronectin to drive lung cancer aggressiveness [66]. In ovarian cancer studies, fibronectin signaling through α5β1-integrin stimulated FAK and Src FAK/Src signaling through c-Met, resulting in cell proliferation [67]. Cells treated with cellular fibronectin showed reduced DNA fragmentation and caspase 3/7 expression, indicative of a reduced degree of apoptosis [68]. In summary, cellular fibronectin can rapidly proliferate and immortalize cells in malignant tumors. In a previous study, the expression of fibronectin 1 was found to be high in the advanced stages of ovarian cancer 34093898. Inhibiting the expression of fibronectin inhibited the growth of breast cancer cells [69]. Stable knockout of the B7-H3 gene in MGC-803 and MKN-45 gastric cancer cell lines has been shown; B7-H3 is a type I transmembrane protein that affects the PI3K/AKT signaling pathway by regulating fibronectin to inhibit gastric cancer cell apoptosis [70]. In a mouse model, inhibition of miR-1278 increased fibronectin 1 expression and promoted gastric cancer progression [71]. Most pieces of evidence show that increased fibronectin expression promotes tumor development, but our results show that increased MCRS1 expression is negatively correlated with fibronectin. However, most theories suggest that fibrin promotes tumor growth and metastasis. However, some studies have shown that it can inhibit tumor growth [72,73,74]. In non-small cell lung cancer, MCRS1 binds to the miR-155 promoter, regulates miR-155, promotes the expression of EMT protein, and increases invasiveness and metastasis of tumors [23]. Although EMT protein and CD31 expression have been reported separately in cancer, no relationship between MCRS1 and EMT protein or CD31 has been established in gastric cancer. In this study, we found that high MCRS1 expression increases fibronectin and CD31 expression. The detailed mechanism underlying the relationship between MCER1 and CD31 is an interesting topic and should be investigated in detail. Our results are more consistent with this theory, but require further clarification.

In research on many anti-cancer drugs, the MAPK/mTOR/p70S6K and Akt pathways or the PI3k-Akt pathway are involved in the regulation of pAKT expression and contribute to inhibition of tumor growth [75,76,77,78]. pAMPK is also one of the proteins involved in proliferation and apoptosis of gastric cancer cells. The use of anti-cancer drug therapy has shown that a decrease in pAMPK expression suppresses tumor growth [79,80,81]. pAMPK is also used as an independent prognostic marker of cancer therapy and effectively predicts the 3-year relapse-free rate of patients [82,83].

Interestingly, in our study, pErk demonstrated a negative correlation with high MCRS1 expression. The gene encoding pErk is a downstream signaling gene in the ERK/MAPK signaling pathway, which regulates cellular biological functions such as cell proliferation, differentiation, cell cycle, apoptosis, and tissue formation, and is also associated with tumor growth [84]. The ERK/MAPK pathway promotes cell proliferation and has anti-apoptotic effects [85]. Inhibition of the ERK/MAPK pathway has been shown to exert an inhibitory effect on tumor cell growth in diffuse large B-cell lymphoma cell lines, as well as in colon cancer cells [86,87,88]. Our results show that pErk negatively correlates with high MCRS1 expression, in contrast to the findings of previous studies. A comprehensive understanding of the pathways related to MCRS1 will aid in evaluating the potential of MCRS1 or MCRS1-targeting agents as candidate drugs for inhibiting tumor growth and improving patient survival.

We performed immunostaining on human tumor samples, but there are still limitations, and animal and cell experiments will be performed in future studies. Recently, an increasing number of studies have used mice and various inhibitors to observe tumor growth. Adding miR-10b-5p to gastric cancer cells and implanting them subcutaneously in mice inhibits the proliferation and migration of gastric cancer cells due to the upregulation of Tiam1 [89]. Mice overexpressing Hpa2 for gastric cancer cells produced smaller tumors and survived longer than control mice [90]. This mechanism is related to the increased phosphorylation of AMP-activated protein kinase (AMPK), a protein that is located in tumor suppressors. In addition to the pre-treatment of cells with inhibitory drugs, the authors also experimented with oral drugs. Oral administration of glutamine significantly inhibited tumor growth in mice and reduced tumor tissue weight. Immunostaining revealed that the PCNA index decreased, which again proved that glutamine could indeed inhibit tumor growth. GSH enhances immune function and activates apoptosis, and GLN significantly increases the activities of Caspase-3, Caspase-8, caspase-9, and PARP [91].

MCRS1 or MSP 58 have rarely been studied in gastric cancer. In 2016, Cui et al. used the gastric cancer cell lines MGC803, BGC823, and NCI-N87 to study MCRS1 expression. Western blotting confirmed that the expression of MCRS1 in gastric cancer cell lines was higher than that in normal cells [21]. Western blot results of gastric cancer cells are similar to our results from immunostaining of human gastric cancer tissues, but the exact mechanism remains unclear. In addition to surgery, several tumor-growth-inhibiting drugs have been developed for treating gastric cancer (e.g., miR-10b-5p, Hpa2, and our previously proposed high mobility group A1) [92]. None of the MCRS1s explored in this study had the potential to inhibit cancer growth.

High expression of MCRS1 in gastric cancer increases the expression of Ki-67, Caspase3, CD31, fibronectin, pAKT, and pAMPK; therefore, MCRS1 positively correlates with these proteins. We believe that these findings are related to tumor proliferation, metastasis, invasion, or infiltration, but the precise mechanisms are still unknown and need to be clarified. In the future, we hope to use cell experiments or animal experiments to further understand and establish the relationships between MCRS1 and these proteins. In the future, after clarifying the relevant mechanism, we will try to use MCRS1 as a target to inhibit and develop related drugs for the treatment of tumor growth.

There are still many limitations in this study because the tumors have been prepared as paraffin samples and there is a need for clinical diagnostic testing. We were unable to obtain more samples for further analysis, such as molecular biology experiments, Western blotting and real time PCR. Although we analyzed the expression of phosphorylated markers such as pAKT, pErk, pSTAT3 and pAMPK, which are related to the level of MCRS1 expression, unfortunately we cannot do more analysis to compare the ratio of unphosphorylated markers to those after phosphorylation. We will plan a complete experimental plan in the future, obtain fresh tumor tissue for analysis, and do our best to analyze the above phosphorylation pathway completely.

In conclusion, the high expression of MCRS1 in gastric cancer was positively correlated with Ki-67, Caspase3, CD31, fibronectin, pAKT and pAMPK. The expression of MCRS1 was high, while the expression of pErk was low. The expressions of MCRS1 and pErK were negatively correlated. The high expression of MCRS1 increased the risk of death by 2.44 times compared with the low expression group at the same time after suffering from gastric cancer. The underlying mechanisms need further study and a larger patient sample is also mandatory in the future validation.

## Figures and Tables

**Table 1:** Antibodies used for immunohistochemical staining.

Antibody	Brands	Catalog Number	Proportion
MCRS1	Sigma-Aldrich	HPA039057	1:400
Ki-67	BioLegend	350503	1:100
Caspase3	Cell Signaling	9664	1:100
CD31	Abbiotec	250590	1:500
E-cadherin	Abcam	ab40772	1:100
N-cadherin	Abcam	ab76011	1:100
Fibronectin	Santa Cruz	SC-8422	1:50
pAKT	GeneTex	GTX11901	1:50
pERK	R&D	AF1018	1:200
pSTAT3	Abcam	ab76315	1:50
pAMPK	Cell signal	2535	1:100

pAKT, phosphorylated-protein kinase B; pERK, phosphorylated-extracellular signal-regulated kinase; pSTAT3, phosphorylated signal transducer and activator of transcription 3; pAMPK, phosphorylated-activated protein kinase.

**Table 2:** Demographics of patients with high and low MCRS1 expression.

		Low MCRS1 Expression	High MCRS1 Expression	Total	*p*-Value
Age (years)	<65	45 (90.0%)	5 (10.0%)	50 (27.6%)	0.586
	≥65	114 (87.0%)	17 (17.0%)	131 (72.4%)	
Gender	Female	106 (86.9%)	16 (13.1%)	122 (67.4%)	0.572
	Male	53 (89.8%)	6 (10.2%)	59 (32.6%)	
Differentiation	Well	48 (92.3%)	4 (7.7%)	52 (28.7%)	0.586
	Moderate	111 (86.0%)	18 (14.0%)	129 (71.3%)	
Stage	I and II	67 (90.5%)	7 (9.5%)	74 (40.9%)	0.352
	III and IV	92 (86.0%)	15 (14.0%)	107 (59.1%)	

**Table 3:** Correlation of MCRS1 with common tumor proliferation and invasion markers and the expression level of MCRS1 in the H-Score of different biomarkers. (* *p*-Value < 0.05; ** *p*-Value < 0.01; *** *p*-Value < 0.001).

Biomarkers	MCRS1 Low Expression	MCRS1 High Expression	R	*p*-Value
Ki-67	22.88 (31.08)	39.29 (55.32)	0.439	<0.001 ***
Caspase3	6.18 (3.55)	9.37 (4.70)	0.270	<0.001 ***
CD31	25.63 (21.26)	24.34 (18.49)	0.271	<0.001 ***
Fibronectin	59.73 (98.11)	33.70 (46.6)	−0.187	0.001 **
pAkt	17.87 (25.43)	14.97 (12.30)	0.281	<0.001 ***
pErk	9.02 (3.21)	11.34 (0.82)	−0.121	0.029 *
pSTAT3	2.80 (0.83)	5.59 (0.16)	0.051	0.358
pAMPK	4.89 (3.42)	10.42 (5.96)	0.182	0.001 **

**Table 4:** Hazard ratios of MCSR1. (** *p*-Value < 0.01).

		Hazard Ratio (95% CI)	*p*-Value
MCRS1 expression	Low	Reference	0.005 **
	High	2.44 (1.31–4.57)	
Age (years)	<65	Reference	0.184
	≥65	0.70 (0.41–1.18)	
Gender	Female	Reference	0.311
	Male	0.74 (0.42–1.32)	
Differentiation	Moderate	Reference	0.079
	Well	1.74 (0.94–3.34)	
Stage	I and II	Reference	0.001 **
	III and IV	2.50 (1.44–4.35)	

## Data Availability

The data presented in this study are available upon request from the corresponding author. The data are not publicly available because they still need to be protected, so they are not provided.
